# Changes in diet and physical activity resulting from the Shape Up Somerville community intervention

**DOI:** 10.1186/1471-2431-13-157

**Published:** 2013-10-04

**Authors:** Sara C Folta, Julia F Kuder, Jeanne P Goldberg, Raymond R Hyatt, Aviva Must, Elena N Naumova, Miriam E Nelson, Christina D Economos

**Affiliations:** 1 John Hancock Research Center on Physical Activity, Nutrition, and Obesity Prevention, Friedman School of Nutrition Science and Policy, Tufts University, 150 Harrison Ave, Boston, MA 02111, USA; 2 Department of Public Health and Community Medicine, School of Medicine, Tufts University, Medford, USA; 3 Department of Civil and Environmental Engineering, School of Engineering, Tufts University, Medford, USA

**Keywords:** Eco-social model, Childhood obesity, Diet, Physical activity, Screen time

## Abstract

**Background:**

The purpose of this study is to describe the behavioral changes in children resulting from Shape Up Somerville (SUS), a community-based, participatory obesity prevention intervention that used a multi-level, systems-based approach. It was set in Somerville, an urban, culturally diverse community in Massachusetts, USA.

**Methods:**

This was a non-randomized, controlled 2-year community-based intervention trial with children enrolled in grades 1 to 3 (ages 6-8 years). Overall, the SUS intervention was designed to create environmental and policy change to impact all aspects of a child’s day. Pre-post outcomes were compared between Somerville and two control communities that were chosen based on socio-demographic similarities. Behavioral outcomes were fruit and vegetable and sugar-sweetened beverage consumption; number of organized sports and physical activities per year; walking to and from school; screen and television time; television in bedroom; and dinner in room with television on. These measures were assessed by parent/caregiver report using a 68-item Family Survey Form. Data were analyzed using multiple linear regression, accounting for covariates and clustering by community.

**Results:**

Intervention group children, compared to the control group, significantly reduced sugar-sweetened beverage consumption (-2.0 ounces per day; 95% CI -3.8 to -0.2), increased participation in organized sports and physical activities (0.20 sports or activities per year; 95% CI 0.06 to 0.33), and reduced their screen time (-0.24 hours per day; 95% CI -0.42 to -0.06).

**Conclusions:**

Results of this study, particularly intake of sugar-sweetened beverages and screen time, are similar to others that used a multi-level approach to realize change in behavior. These results support the efficacy of a multi-level and systems-based approach for promoting the behavioral changes necessary for childhood obesity prevention. This study is registered at ClinicalTrials.gov as NCT00153322.

## Background

In the United States, few children are engaging in the diet and physical activity behaviors that would promote health and prevent obesity. The majority of children ages 4-18 years fail to meet current recommendations for fruits and vegetables, with less than 10% of children meet vegetable recommendations [1]. On the other hand, low-nutrient, energy-dense foods, including sugar-sweetened beverages, comprise nearly 40% of children’s daily caloric intake [2]. Although no national surveillance system exists to track physical activity of children younger than adolescence, accelerometer data indicate that less than half of children ages 6-11 years are getting at least 60 minutes per day of physical activity, as recommended [3]. Sedentary behaviors such as television viewing and computer and video game use are also associated with obesity in children [4-7]. The American Academy of Pediatrics recommends limiting children’s total media time to no more than 1 to 2 hours of quality programming per day and removing television sets from children’s bedrooms [8]. Yet children ages 8-18 years watch an estimated 4.5 hours per day of television shows, use a computer and video games for nearly three hours, and 70% have a television in their bedroom [9].

A community-wide approach to childhood obesity prevention has the potential to be more effective, since it accounts for the range of social and physical contexts that help shape behavior [10]. Another advantage is that the burden for change does not fall disproportionately on any one sector. Instead, modest, low cost, and replicable changes can be made in each of the settings within the community. The broader focus also has the potential to reach a larger proportion of the population [11]. Sustainability is more likely through environmental and policy changes, and the potential for community ownership that translates into institutional and cultural changes. This approach is consistent with the Social Ecological Model in which multiple spheres of influence (individual-interpersonal-organizational-community-public policy) are targeted [12], and further with systems theory that recognizes the dynamic interplay within and among these levels [13]. The feasibility and effectiveness of a multi-level, multi-setting strategy has now been demonstrated in several childhood obesity prevention studies [14-22].

Shape Up Somerville (SUS): Eat Smart, Play Hard was conducted in Somerville, MA during 2002-2005. It was one of the first projects to take a social ecological and systems approach, using community-based participatory research (CBPR) principles [23,24], to address obesity prevention in elementary school-aged children. It resulted in a significant reduction in BMI z-score in the intervention community compared with controls [15,25]. SUS was designed to create changes in multiple environments within the community, among them the before, during, and after school environments. These changes were supported through additional changes within the home and broader community environments. They were designed to increase availability of foods of lower energy density, with an emphasis on fruits and vegetables; to discourage foods high in fat and sugar, including sugar-sweetened beverages; and to increase the opportunities for physical activity.

The purpose of this analysis is to assess the changes in children’s diet, physical activity, and sedentary behaviors that occurred during the SUS intervention. By better understanding the specific behaviors that changed, we gain insight into which environmental and policy changes were likely to have been effective in this community-wide intervention.

## Methods

### Study design

SUS has been described in detail elsewhere [15]. Briefly, it was conducted as a non-randomized, controlled intervention trial that included 3 communities: Somerville and two control communities in Massachusetts. The main intervention period took place over one school year (Fall 2003 through Spring 2004). In the second year of the intervention (through Spring 2005), ownership of the intervention was transferred to the community, with most activities implemented by community leadership. This was by design, to understand the potential for sustainability in this type of project. The results presented here are for the entire 2 intervention years of the study, Fall 2003 (Pre) through Spring 2005 (Post).

### Setting

Somerville was chosen as the intervention community because of our established and on-going relationship with that community, as recommended for successful CBPR projects [23,24]. Potential control communities in Eastern Massachusetts were identified based on available U.S. Census data. Communities were considered matches if they had similar community demographic characteristics such as non-English speaking in the home (28-36%), median household income ($39,507-$46,315), and percentage living below the poverty level (12.5-14.5%) [26,27]. The first two communities that provided written commitment to participate were chosen as controls.

### Participants

**Figure 1:**
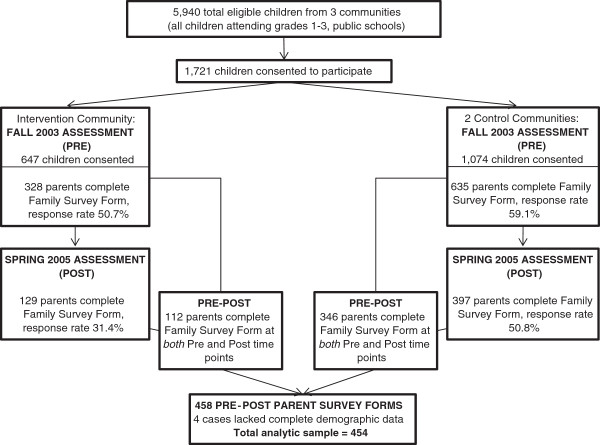
Study flow and assessment points of data used to analyze behavioral outcomes during the 2-year Shape Up Somerville intervention period.

The target population for the intervention was all children enrolled in grades 1 to 3, typically ages 6 to 8 years, in the 10 Somerville public elementary schools. Figure 1 depicts the flow of participants through the study. Behavioral outcomes were assessed by parent/caregiver report through the Family Survey Form. In Somerville, 328 parents completed the form, a 50.7% response rate among those who had consented their children. In the two control communities, 635 parents completed the form, a 59.1% response rate among those consented. At the end of the two-year intervention, 458 parents/caregivers had complete pre-post data: 112 of those in Somerville and 346 of those in the control communities. Four lacked complete demographic data, and therefore a sample of 454 was used in the analysis.

All aspects of the study were approved by the Institutional Review Board at Tufts University. Parental written consent and assent by children over age 7 years were required for study inclusion.

### Intervention

The goal of SUS was to influence multiple aspects of an elementary school child’s day (see Economos et al. 2007 [15] and http://nutrition.tufts.edu/research/shapeup). In the before-school environment, the pre-existing free breakfast program was changed to increase the fresh fruit, low-fat milk, and whole grains served; the breakfast program also incorporated taste tests and adult coordinators, who supervised the meal and modeled healthy eating [28]. A Walk to School Campaign was launched that included a “walking to school bus”, traffic calming tactics, walking contests, and maps highlighting safe routes to school.

In the school environment, changes to school lunch were realized through a strong collaboration with food service [28]. These changes included highlighting a fruit and vegetable each month, with taste tests; educational posters and tabletop tents around the cafeterias; new kitchen preparation and serving equipment and training for food service staff, so that more fresh fruits and vegetables could be served; and healthier a la carte snacks. A classroom curriculum was implemented that included a weekly 30-minute nutrition and physical activity lesson. Recess was enhanced with new play equipment and active play game cards, and a school wellness policy was developed.

In the after school environment, a curriculum was also implemented that included 28 lesson plans that used crafts, cooking demonstrations, and physically active games as vehicles for education. A Walk from School Campaign was launched, using the same strategies as the Walk to School Campaign.

The home environment was targeted through parent outreach and education, including bi-monthly newsletters, nutrition forums, and family events, and a child’s Health Report Card that was mailed to parents/caregivers each year. Activities in the larger community included a restaurant initiative [29] that involved working with restaurants across the city to enhance food options by offering more low-fat dairy products, offering some dishes in smaller portion sizes, offering more fruits and vegetables as side dishes, and having visible signs that highlighted the healthier options. Other community activities included trainings for local physicians on approaching and counseling families with an overweight or obese child; development and dissemination of community resource guides that were posted on school and city websites and updated annually; regular local media placement, including a monthly column in a city newspaper; and the development of community-wide policies, including a comprehensive Wellness Policy.

We conducted extensive process evaluation to document the extent of implementation of all activities during the study [15]. Activities, which were developed with community input, were for the most part implemented as intended.

### Outcome measures

A 68-item Family Survey Form was mailed to parents/caregivers along with a postage-paid return envelope during the Pre (Fall 2003) and Post (Spring 2005) periods. A reminder postcard was sent if the survey was not returned within 2 weeks. Non-responders were sent a second survey 1 week later. All households received the survey in English. In addition, based on the primary language spoken in the home, 10.9% of families received the survey in Portuguese, 3.4% in Haitian Creole, and 11.0% in Spanish.

Parents/caregivers reported the number of servings of fruits eaten by their children in a typical day (“0” to “5 or more”). A serving was described as about the size of a medium apple or 1 cup of melon cubes. They likewise reported servings of vegetables, with a serving described as about a cup of leafy vegetable or a half cup of cooked vegetable like carrot or potato. Parents also reported the number of 12-ounce cans of soda (such as Mountain Dew, Coca Cola, Pepsi) and other sugar sweetened beverages (Hi-C, Kool-aid, sport drinks) their child drank per day or per week.

Parents/caregivers listed the organized sports and physical activities (lessons and/or teams) that their children participated in during each season over the past year, and reported the number of times their children walked to and from school during a typical school week (0 to 5 days). The amount of time, in hours and minutes, that children spent watching TV, watching videos or DVDs, playing video games, and playing on the computer on a typical school day and weekend day was captured using questions in a standard, validated format [30]. Parents/caregivers also reported how often their child ate dinner in a room with the TV turned on (response choices: a lot, sometimes, not very much, never, and don’t know) and whether or not there was a TV in the room where the child sleeps (yes/no).

Parents/caregivers were asked to report their own demographic and anthropometric information on the Family Survey Form, including education, age, ethnicity, marital status, and height and weight. They were also asked to indicate whether there were household rules related to television and computer use, bedtime, consumption of sugary foods and beverages, and snacking. Basic child demographic information (sex, birth date, grade, race/ethnicity, and primary language at home) was collected from parents/caregivers by a separate questionnaire at the time informed consent was obtained.

### Statistical analysis

To describe the analytic sample at baseline, we performed independent t-tests or χ2 tests to examine differences between the intervention and control communities. We used multiple linear regression models (one model for each outcome) to assess pre-post behavior change comparing the intervention and control communities. Since subjects were not randomly assigned to treatment groups, the analytic approach accounted for the intraclass correlation resulting from similarities among subjects within the same community. Since there were so few clusters, we did not use a multilevel modeling approach, but instead used a survey procedure (PROC SURVEYREG; SAS Institute, Inc., Cary, NC) to account for the intra-class correlation between communities, the primary sampling unit [31]. For dichotomous and categorical outcomes, we applied the same estimating procedure using a cluster statement in STATA (Version 9, StataCorp LP). Because no other interventions were introduced in either control community during the study period, we pooled the two control communities as planned a priori. The covariates that were included in all models were sex, age, race/ethnicity, primary language at home, and baseline values of the outcome. Any other variables that correlated significantly with an outcome were also included in the model. Sample sizes differed for each of the analyses since data were missing in a non-uniform way for each of the covariates. We also conducted the analysis after accounting for missing data using multiple imputation; results did not differ significantly and the original analysis is reported. The alpha-level was set at p < 0.05 for all analyses.

## Results

Characteristics of the sample at baseline are shown in Table 1. There were no significant differences between groups in gender distribution, weight category, primary language spoken in the home, parent foreign born status, or number of household rules related to television and computer use, bedtime, consumption of sugary foods and beverages, snacking, and hand washing. However, the intervention group children were slightly older than the comparison group (7.7 years versus 7.4 years), with a corresponding lower percentage in the first grade. The groups also differed significantly on race/ethnicity (68.5% of intervention group children were white, compared to 56.9% of control group children). Intervention group children also had a higher percentage of parents/caregivers that were married (80.9 versus 67.0%), fewer siblings (mean 1.3 versus 1.6), and their mothers had a lower self-reported mean BMI (24.3 versus 26.4).

**Table 1:** Child and parent/household baseline characteristics of analytic sample by intervention status

	**Controls**	**Intervention**
**Child characteristics**		
	n = 343	n = 111
Age (y)*		
Mean (SD)	7.4 (0.9)	7.7 (1.0)
Gender		
Male	46.9%	47.7%
Female	53.1%	52.3%
Grade*		
One	41.1%	29.7%
Two	28.3%	39.6%
Three	30.6%	30.6%
Ethnicity*		
White	56.9%	68.5%
Black	11.7%	8.1%
Hispanic	10.8%	4.5%
Asian	2.6%	11.7%
Other	18.1%	7.2%
Weight Category^1^		
< 85th percentile	63.7%	61.2%
85^th^ - 95^th^ percentile	16.2%	19.4%
> 95^th^ percentile	20.1%	19.4%
**Parent/Household characteristics**		
Married*	67.0%	80.9%
US Born Mom and/or Dad		
both foreign	24.7%	24.3%
1 US	9.9%	15.0%
2 US	65.4%	60.7%
Primary Home Language		
English	85.1%	84.7%
Spanish	6.4%	2.7%
Creole	1.5%	0.9%
Portuguese	5.0%	8.1%
Other language(s)	2.0%	3.6%
# Siblings, Mean (SD)*	1.6 (1.2)	1.3 (1.0)
Mom BMI, Mean (SD)*	26.4 (6.2)	24.3 (4.6)
# Rules, Mean (SD)	0.8 (0.2)	0.8 (0.2)

*Control group differs significantly from intervention group, p < 0.05.

^1^Percentile rankings from Centers for Disease Control and Prevention BMI-for-age growth charts (for either girls or boys).

Both groups fell short of meeting behavioral recommendations at baseline (Table 2). Neither group consumed five or more fruits and vegetables per day. Both groups viewed more than the recommended two hours or less of screen time per day. A television in the bedroom is not recommended, yet in Somerville, 29.4% of children had one in their bedroom, and in the control communities, 50.4% of children did. Although sugar-sweetened beverages should be minimized or eliminated [32], parents/caregivers in both groups reported that their children consumed about half of a 12-ounce can per day.

**Table 2:** Adjusted differences in behaviors between intervention and combined control communities after 2-year intervention period

	**Baseline**				**After intervention**				**Pre–post change: adjusted difference**^**1**^		**Model properties**	
**Behavior**	**Control group**		**Intervention group**		**Control group**		**Intervention group**					
	n	Mean (SD)	n	Mean (SD)	n	Mean (SD)	n	Mean (SD)	Effect (95% CI)	p-value	additional covariates	R^2^
Fruit & vegetable (servings/day)	317	3.1 (1.5)	103	3.5 (1.6)	317	3.4 (1.6)	103	3.7 (1.8)	0.16 (-06,0.38)	0.09	parental foreign born status, # siblings	0.18
Sugar-sweetened beverages (ounces/day)	265	6.5 (6.0)	72	6.1 (6.3)	265	7.6 (7.0)	72	5.5 (6.7)	-2.00 (-3.76,-.25)	0.04	# rules	0.21
Sports (# per year)	343	2.9 (2.8)	111	3.6 (2.9)	343	3.4 (2.7)	111	4.0 (2.9)	0.20 (0.06,0.33)	0.02		0.21
Walk to/from school (# trips per week)	248	2.7 (4.0)	87	3.5 (4.1)	248	2.6 (3.9)	87	3.9 (4.2)	0.65 (-0.53,1.82)	0.14	parent marital status, maternal BMI	0.20
TV time (hrs/day)	325	2.2 (1.1)	104	1.6 (1.1)	325	2.2 (1.0)	104	1.7 (1.2)	-0.24 (-0.51,0.04)	0.06	# rules	0.27
Total screen time (hrs/day)	332	3.8 (1.8)	106	2.7 (1.6)	332	3.9 (1.9)	106	3.0 (2.2)	-0.24 (0.42,0.06)	0.03	parent marital status, # siblings, # rules	0.22
TV in bedroom^2^ (% yes)	250	50.4%	85	29.4%	250	54.8%	85	31.8%	0.39 ( 0.11,0.89)	0.13	child weight category, # siblings, maternal BMI	--^4^
Dinner with TV^3^ (% not very much/never)	337	61.4%	110	73.6%	337	62.0%	110	71.8%	OR 0.94 (0.88, 1.00)	0.06		--^4^

^1^All models adjusted for sex, age, ethnicity, primary language in the home, baseline value, and clustering by community. Additional covariates that were unbalanced between study groups, which demonstrated significant correlation with outcomes are also added to the model. These included parent marital status, parental foreign born status, number of siblings, maternal BMI, and rules.

^2^In the regression model to obtain the intervention effect, the dependent variable is a 3-level ordinal variable: -1 (acquired a TV in bedroom), 0 (no change), 1 (TV removed from bedroom).

^3^The outcome is dichotomous. Adjusted difference is reported as an odds ratio. OR < 1 indicates that those in the intervention were less likely to watch TV with dinner not very much/never at the end of 2 years.

^4^R-squared values were not calculated for the ordinal or dichotomous variables.

To understand how the analytic sample of Family Survey Form responders (n = 454, see Figure 1) compared to the overall SUS study population, we compared it with the total sample of consented children (total n = 1,694: 647 intervention and 1,074 control children, see Figure 1) with baseline demographic data. The analytic sample did not differ from the overall sample based on child’s age, gender or grade. However, a significantly higher proportion of the analytic sample was white (59.7% versus 42.3%) and spoke English as the primary language in the home (85.0% versus 70.8%).

The intervention had a significant effect on sugar sweetened beverages (-2.0 ounces per day; 95% CI -3.8 to -0.2), the number of organized sports and physical activities (0.20 sports or activities per year; 95% CI 0.06 to 0.3), and overall screen time (-0.24 hours per day; 95% CI -0.42 to -0.06) (Table 2). The intervention effect was favorable but non-significant for all other behaviors except eating dinner with the TV on, which was unfavorable but non-significant.

## Discussion

This study helps elucidate some of the behavior changes in children that resulted from Shape Up Somerville, one of the first multi-level, multi-setting community-based childhood obesity prevention interventions [15]. Baseline data indicate that there was a great need for effective programming to help children meet behavioral recommendations related to obesity prevention. Compared to controls, children in the Somerville intervention decreased sugar sweetened beverages by more than a 12-ounce can per week compared to controls and increased their participation in organized sports and activities by 0.2 per year, a slight but significant effect. They decreased their overall screen time by nearly 15 minutes per day. These behavioral changes likely helped contribute to the effect on BMI z-score that was observed in the Shape Up Somerville project [15,25], as they would have helped reduce the energy gap between calories consumed relative to calories burned over and above those needed for normal growth and development by approximately 56 calories per day (a decrease in intake of approximately 144 kcals per week from sugar-sweetened beverages; and an increased expenditure of approximately 250 kcal per week from sports participation and from replacing screen time with moderate activity [33]).

We did not observe an intervention effect on fruit and vegetable consumption. Other studies published to date that used a multi-level, social ecological approach collectively indicate that this approach can be effective at increasing fruit and vegetable consumption whether the primary target is the home, school, or community environments [16,19,34,35]. However, parent/caregiver report was likely to capture consumption that took place mainly in the home, and may have missed changes in other environments. The amount of fruits and vegetables served at school lunch did increase significantly [28], for example.

The SUS intervention resulted in a decrease of 2 ounces of sugar-sweetened beverage per day. In addition to targeting individual behavior change through the in-school and after school curricula, environmental changes limited the availability of sugar-sweetened beverages through enactment of the wellness policy, which required beverages provided for snack in the classroom, sold as a la carte snacks, or sold for fundraisers to meet nutritional guidelines that limited sugar content. In addition, the home environment was targeted through parent nutrition forums and newsletters that raised awareness about the potential health detriments caused by sugar-sweetened beverages. In the community environment, restaurants were required to offer low-fat dairy as an alternative to sugar-sweetened beverages to become a Shape Up Somerville approved restaurant [29]. The APPLE intervention [36], that likewise used a multi-level approach included curriculum lessons highlighting the negative effects of sugar-sweetened beverages, increased availability of water at schools, and provision of a community-wide healthy eating guide. APPLE resulted in a decrease in intake of carbonated beverages at 2 years [16]. This suggests that this behavioral target is a particularly feasible for modification in this type of intervention.

There was no significant intervention effect on active transport to and from school, despite substantial efforts to encourage it. Parents indicated that safety concerns were a major barrier in the formative phase of the study. Efforts to address this included the institution of walking school busses, traffic calming tactics, repainting of cross walks, and creation of maps highlighting safe routes to school. Walking was promoted through walking contests and the observance of International Walk to School Day. It appears that substantial environmental change along with awareness campaigns were insufficient to address the major barrier of safety concerns, perhaps because of the young age of the children and the highly urban environment.

We observed a significant intervention effect on the number of organized sports and physical activities per year, such as lessons and teams that children participated in. Those most frequently reported were swimming, dance, and soccer. The increase is notable since it requires a community-wide approach: more programming must be available, availability must be communicated, and barriers to participation must be removed. The intervention included a built environment training that emphasized the importance of a safe and accessible environment with good programming. It also included trainings by the SUS taskforce that worked throughout the intervention period to develop and implement a wellness policy that included increased activity opportunities. Finally, this result suggests that the Physical Activity Resource Guide was useful, and that parent/caregiver outreach efforts were successful.

Shape Up Somerville children decreased their overall screen time by nearly 15 minutes per day compared to children in the control communities. For this behavioral target, effectiveness may have been achieved through the consistent messaging that children received from their parents, teachers, after school staff, doctors, the mayor (who was a key “community champion”), and other influential adults within the community. An effect on screen time was similarly observed in the Switch [19] and Travis County CATCH [35] studies. This suggests that messaging implemented at multiple levels may create synergistic effects that positively influence children’s screen-related behaviors.

We did not find an intervention effect on whether a child had a television in the bedroom or not, or whether the family ate dinner in a room with the television on. Intervention components focusing more intensively and specifically on the home/family environment may be necessary to achieve change in these outcomes.

This study has a number of potential limitations. Implementation using CBPR required leveraging an established relationship with the target community. For that reason, Somerville was chosen for the intervention rather than being randomized. However, control communities were chosen to match Somerville closely based on demographic characteristics. The presence of controls helps rule out the possibility that the observed changes were the result of secular trends alone. It is also possible that the results are not generalizable to other communities. However, Somerville is a diverse urban community that had access to a typical level of resources. The intervention was designed to be flexible and to operate through settings that would be common to any community.

The behavioral outcomes were measured by parent/caregiver report, limiting the ability to capture changes that may have occurred outside the home environment. Furthermore, it raises the issue of recall bias. In particular, awareness and buy-in to the intervention may have caused parents/caregivers of children in the intervention group to perceive and report greater changes in their children’s behaviors than actually existed. While this cannot be ruled out, significant effects were not realized for all outcomes; and in fact outcomes that were heavily emphasized in the intervention, such as walking to and from school, were not significant. Recall bias due to straightforward memory inaccuracies would be expected to be similar in both control and intervention groups.

Although the intervention was designed to target the entire community, only a subset of children in Somerville and the control communities were measured and followed, and fewer still had parents/caregivers who provided complete data for the 2 years of the intervention. In all communities, enrollment in the overall study was limited by the requirement for parental informed consent in diverse communities with many languages spoken and a lack of familiarity with research. The analytic sample of parents who completed the Family Survey Form pre and post-intervention was likely further hindered by this issue, as suggested by the fact that a higher percentage of Family Survey Form responders spoke English as the primary language, despite all parents/caretakers receiving the survey in their primary language. It is possible that those who responded to study recruitment were more interested in diet and physical activity behaviors, and were already practicing healthier behaviors at baseline. For this reason, true changes may have been more difficult to detect. Finally, a limitation is that this study describes data collected in 2003 and 2005. However, the targeted behaviors remain on the national agenda since children continue to fail to meet recommendations. The social ecological and systems-based approach taken in this study remains highly relevant for childhood obesity prevention, and as more communities are taking this approach it is important to gain an understanding of the complex systems in place and the impact on behavior.

Despite these limitations, this study provides evidence for change in several targeted behaviors that was sustained over a two-year period, which included the transition from a researcher/community partnership in the first year to the community alone during the second year. The City of Somerville has continued and expanded many of the initiatives, and community-generated data suggest that children’s weight status outcomes have continued to improve in the years since the original intervention [37]. These results therefore suggest success in building community capacity and support the efficacy of a social ecological and systems-based approach for promoting sustainable change for childhood obesity prevention.

## Conclusions

Although a growing body of literature supports the multi-level, multi-setting community-based approach, further studies are needed to confirm its efficacy and generalizability. Future studies may expand this work by incorporating a wider variety of behavioral measures. Studies may also consider best practices for engaging community leaders in public health activities.

These results, within the context of the overall SUS intervention, suggest several strategies for practitioners. SUS was designed to create synergies to ensure that opportunities for healthier eating and increased physical activity occurred in multiple settings. These synergies were fostered in a number of ways, including provision of professional development on nutrition and physical activity to all school staff; involving community champions, including the mayor and other city employees; training local physicians and clinical staff; and involving many stakeholders in community events and local media.

It is particularly notable that the initiatives transitioned successfully to the City of Somerville, utilizing additional grant funding and city resources to make low-cost changes across multiple sectors so that all residents continue to benefit, regardless of income. This strategy therefore has potential to help reduce income-based health disparities, with the entire community supporting the growth of all children into healthy adolescents and adults.

## Abbreviations

SUS: Shape Up Somerville; CBPR: Community-based participatory research; BMI: Body mass index; APPLE: A pilot programme for lifestyle and exercise; CATCH: Coordinated approach to child health.

## Competing interests

The authors declare that they have no competing interests.

## Authors’ contributions

SCF and JK were responsible for the primary analysis, in consultation with RRH. All authors participated in the design of the study, the analysis plan, and interpretation of results. All authors have read and approved the final manuscript.

## Pre-publication history

The pre-publication history for this paper can be accessed here:

http://www.biomedcentral.com/1471-2431/13/157/prepub
